# Optimizing athletic engagement and performance of obese students: an adaptive approach through basketball in physical education

**DOI:** 10.3389/fspor.2024.1448784

**Published:** 2025-01-28

**Authors:** Oumayma Slimi, Antonella Muscella, Santo Marsigliante, Mourad Bahloul, Georgian Badicu, Abdullah F. Alghannam, Fatma Hilal Yagin

**Affiliations:** ^1^ High Institute of Sport and Physical Education, University of Sfax, Sfax, Tunisia; ^2^ Research Laboratory: “Education, Motricité, Sport et Santé”, EM2S, LR19JS01, High Institute of Sport and Physical Education of Sfax, University of Sfax, Sfax, Tunisia; ^3^ Department of Biological and Environmental Sciences and Technologies (Di.S.Te.B.A.), University of Salento, Lecce, Italy; ^4^ Higher Institute of Education and Continuing Training, Virtual University of Tunis, Tunis, Tunisia; ^5^ Department of Physical Education and Special Motricity, Faculty of Physical Education and Mountain Sports, Transilvania University of Braşov, Braşov, Romania; ^6^ Lifestyle & Health Research Center, Natural and Health Sciences Research Center, Princess Nourah Bint Abdulrahman University, Riyadh, Saudi Arabia; ^7^ Department of Biostatistics and Medical Informatics, Faculty of Medicine, Inonu University, Malatya, Türkiye

**Keywords:** obesity, physical education, basketball, adaptive approach, adolescents

## Abstract

**Introduction:**

Obesity in adolescents is associated with reduced physical activity and athletic engagement, highlighting the need for tailored physical education programs. This study evaluated the effects of a 7-week adapted basketball program on the performance and athletic engagement of students with obesity.

**Methods:**

Sixty-two students with obesity (23 boys, 39 girls, aged 15-17) were randomly assigned to an experimental group (EG, *n* = 30; 11 boys, 19 girls) participating in adapted basketball sessions or a control group (CG, *n* = 32; 12 boys, 20 girls) attending standard basketball lessons. Both groups completed 52-minute sessions twice weekly. Pre- and post-intervention assessments included a questionnaire evaluating perceptions of physical education and athletic performance during final matches.

**Results:**

The EG showed significant improvements in interest, motivation (*p* < 0.05), perceived competence (*p* < 0.001), and reduced exercise difficulty (*p* < 0.001). Perceived fitness levels increased significantly only in EG girls (*p* = 0.013). In contrast, no significant changes were observed in the CG before and after the intervention for any of the parameters. During matches, the EG outperformed the CG, with more successful shots (girls: *p* = 0.0004; boys: *p* = 0.012), fewer missed shots (girls: *p* = 0.033; boys: *p* = 0.046), and more successful passes (*p* = 0.032, *η*² = 0.042).

**Discussion:**

These results demonstrate that adapted physical education programs can serve as effective interventions for improving physical activity and promoting inclusion among adolescents with obesity while also serving as a preventive measure against obesity.

## 1 Introduction

Childhood and adolescent obesity have emerged as significant public health concerns worldwide, prevalent in both developed and low- to middle-income countries, particularly in urban areas (1, 2). Unfortunately, overweight and obesity are associated with various risk factors for cardiovascular diseases, hypertension, type 2 diabetes, and other health issues (2). Obesity stems from an excess accumulation of fat tissue in the body, often due to unhealthy eating habits and a sedentary lifestyle (3, 4). According to the World Health Organization (WHO), a body mass index (BMI) exceeding 25 indicates overweight, while a BMI surpassing 30 signifies obesity (5).

Energy imbalances, resulting from excessive calorie intake (6), inadequate physical activity (7), or both (8), primarily contribute to overweight and obesity. However, obesity is a multifactorial condition influenced by genetic (9), hormonal (10), environmental (11), and socioeconomic factors (12). Genetic predispositions can affect fat storage and appetite regulation, while hormonal imbalances, such as those involving leptin and insulin, can disrupt metabolic processes (13). Environmental influences, such as limited access to healthy foods and safe places for physical activity, play a significant role (14). Socioeconomic factors further impact obesity risk, as individuals in lower socioeconomic strata may face additional barriers to maintaining a healthy weight (15).

Acknowledging these various contributing factors provides a more comprehensive understanding of obesity and underscores the need for multifaceted interventions. Recent systematic reviews and meta-analyses support this broader perspective by addressing these multiple influences on obesity. Childhood overweight or obesity frequently precedes metabolic syndrome, compromised physical and mental health, respiratory problems, and glucose intolerance, often persisting into adulthood (16). Approximately 60% of children with overweight or obesity exhibit at least one additional risk factor for cardiovascular diseases, such as high blood pressure, elevated lipid levels, or increased insulin levels (17). Children with overweight or obesity are at an increased risk of developing abnormal lipid profiles (18). The rising prevalence of overweight and obesity among children and adolescents is largely attributed to reduced levels of physical activity observed in today's youth (19–23). Moreover, limited physical activity and childhood obesity are interconnected, as excessive weight can adversely impact motor coordination performance relative to age and gender (24). Addressing the optimization of athletic involvement and performance among students with obesity poses a substantial challenge within the realm of physical education (PE) (25), where traditional approaches may not adequately address the diverse needs of this population (26). Additionally, overweight and inactive children exhibit lower levels of psychological well-being compared to physically active children of normal weight (27), and as they transition into adulthood, they often struggle with persistent weight issues alongside compromised mental health (28). Consequently, numerous studies advocate for the promotion of physical activity and dietary interventions among children to prevent and ameliorate the challenges associated with childhood overweight or obesity (29, 30). Schools serve as pivotal environments for fostering inclusive learning experiences for all children, adolescents, and young adults, irrespective of their diversities (31, 32). Similar to other academic subjects, PE in schools plays a crucial role in both the general and specific development of individuals (9), often correlating with improved academic performance (33, 34). However, unlike other subjects, PE is sometimes perceived as optional by certain students, who may attempt to evade participation using medical exemptions (35). Adolescents grappling with obesity may resort to avoidance tactics akin to those observed in individuals with anorexia, effectively circumventing disciplinary measures. This highlights a disconnect between school dynamics and familial expectations, wherein the school setting often fails to challenge entrenched family patterns (35). In fact, one of the main objectives of PE is the learning of correct and healthy lifestyles, an aim which becomes a necessity above all for students with obesity (36). Given that adolescents with overweight or obesity frequently possess reduced motor skills and physical fitness compared to their peers with normal weight (37, 38), they encounter numerous challenges during PE classes. This results in them experiencing feelings of fatigue, tension, diminished self-esteem, feelings of incompetence, negative body image, and a tendency to avoid exercise (39, 40). Therefore, it's essential for students with overweight or obesity to have a supportive environment, particularly among their peers and educators, where they are free from stigma or negative perceptions (41). As numerous studies have indicated, children and adolescents who are overweight or obese often encounter more difficulties in peer relationships compared to those of normal weight (42, 43). Consequently, in a physical education setting, these individuals may encounter various psychological obstacles that are not typically experienced by their normal-weight peers (44). Specifically, young people with obesity tend to experience feelings of diminished self-esteem and self-imposed limitations, along with experiences of social isolation and stigma; at times, they may perceive the PE environment as unwelcoming, leading to negative impacts on their self-esteem and body image (6, 45). Nevertheless, it is essential for all students, including those with obesity (46), to develop their skills and learn to navigate their environment, while taking into account their individual characteristics. Creating inclusive environments that allow each student to develop their unique potential is crucial (47). Specifically, tailored approaches can support students with obesity in overcoming the specific challenges they face, without assuming these challenges are universal or that all non-obese peers have similar experiences (31). Therefore, it's essential for educators to create a supportive atmosphere for these students through effective strategies that promote integration and encouragement (48). The physical education instructor plays a crucial role in supporting students with physical disabilities to integrate socially (41, 42). Conversely, physical education can be perceived as challenging, especially when repeated failures contribute to feelings of incompetence and frustration (28). Additionally, students who are overweight or obese must develop skills and engage in activities similar to their peers in an environment that caters to their physical abilities while prioritizing safety (38). Therefore, the presence of students with overweight and obesity represents a significant challenge for educators (37). However, despite recognizing this challenge, there is a limited number of studies that address this problem through practical interventions in the field (32, 49). Additionally, while existing research highlights the importance of adapting PE programs to meet the needs of students with obesity and shows benefits such as improved inclusion, empathetic skills, perception of difficulty, and physical enjoyment (16, 24, 40), few studies have specifically investigated how to optimize athletic engagement and enhance performance among students with obesity (31, 50). This gap underscores the need for targeted research into practical strategies that not only foster inclusive environments but also specifically aim to improve athletic engagement and performance. Incorporating an adaptive, basketball-focused approach could offer a promising opportunity to address this gap by aligning the curriculum with the abilities and preferences of these students (21, 22, 41). We hypothesize that an adapted basketball-focused approach could be an effective strategy to achieve such improvements, therefore, the purpose of this investigation was to evaluate the effects of the adapted basketball exercises over 7 weeks on the performance and athletic engagement of students with obesity.

## 2 Methods

### 2.1 Participants and randomization

A total of 62 students (15–17 years old) with obesity participated in this study. These students were enrolled in the 2nd year secondary school of Regueb (Tunisia) and attended 4 different classes. An online statistical power analysis results, obtained from G*Power, showed that a sample size of 62 subjects would be sufficient to identify significant differences.

Sampling procedures targeted girls and boys based on predefined BMI criteria corresponding to obesity status. Inclusion criteria were based on age- and sex-specific BMI percentiles for adolescents, according to World Health Organization (WHO) guidelines, which consider both age and gender to define overweight and obesity status.

Exclusion criteria included failure to submit a signed parental consent form, lack of commitment to participate for the full duration of the study, incomplete questionnaires provided by parents, and inability to perform the physical tests. Additionally, participants with any diagnosed musculoskeletal, neurological, respiratory, or mental health disorders were excluded. This was verified through a medical history questionnaire completed by the parents based on the basic physical examination conducted by a health care professional.

A randomization procedure (a computer-generated list of random numbers using SPSS, version 24) was performed by the principal investigator (O.S, co-author) in order to randomly assign all students to an experimental group (EG, *n* = 30 including 11 boys and 19 girls) or to a control group (CG, *n* = 32; including 12 boys and 20 girls). After this assignment, no significant differences emerged between these two groups regarding the mean values of weight, height and BMI percentiles (*p* > 0.05, as per ANOVA, see Table 1).

**Table 1:** Anthropometric characteristics of participants (mean ± SD).

	Female			Male		
	EG (*n* = 19)	CG CG (*n* = 20)	*p*	EG (*n* = 11)	CG (*n* = 12)	*p*
Age (years)	16.10 ± 0.74	16.20 ± 0.85	0.72	16.15 ± 1.01	16.06 ± 0.70	0.67
Height (m)	1.51 ± 0.37	1.50 ± 0.16	0.78	1.59 ± 0.16	1.6 ± 0.14	0.81
Weight (kg)	69.06 ± 0.61	72.69 ± 0.42	0.13	77.36 ± 3.83	78 ± 5.11	0.11
BMI (kg/m^2^)	32.28 ± 1.35	32.65 ± 1.49	0.64	31.60 ± 1.18	31.64 ± 1.19	0.21
Percentile BMI	>95th	>95th	>0.05	>95th	>95th	>0.05
Classification BMI	Obesity	Obesity		Obesity	Obesity	

Data are espessed as means ± standard deviations. BMI, body mass index; EG, experimental group; GC, control group.

### 2.2 Ethical considerations

The study adhered to ethical standards outlined in the Declaration of Helsinki. Approval was obtained from the local committee at the Higher Institute of Sport and Physical Education of Sfax (049/2022). Ethical measures included respecting students' sensitivity regarding their physical appearance and ensuring minimal discomfort. All procedures were integrated into regular PE classes, with implicit consent obtained through participation. Before the beginning of the study, we conducted informational sessions where students could ask questions and express any concerns regarding the research. This feedback was taken into consideration, and adjustments were made to the study protocol as needed. Participant and their parents received an informative about the study, including an informed consent form that outlined the nature of participation, benefits, and the right to withdraw at any time. This form was designed to ensure that students fully understood their involvement in the study. After the parent's initial approval, we also established a follow-up mechanism to ensure that students remained comfortable with their participation throughout the study. This included periodic check-ins and the option to withdraw at any time, with the option to express any discomfort during the assessments and had trained staff available to address any concerns.

### 2.3 Anthropometric measurements

Height (m) and weight (kg) were measured with a height rod and an electronic scale (Tanita, Tokyo, Japan). Accuracy and repeatability of the measurements are ensured (42), considering that they were carried out by the same operator following the same steps, at the same hours of the day. The body mass index (BMI) was calculated for each subject according to the formula: BMI = body mass/Height^2^ (kg/m^2^). To classify students' weight status, we used age- and sex-specific BMI percentiles for adolescents, in accordance with the World Health Organization (WHO) guidelines. Specifically, a BMI above the 95th percentile for age and sex was used to identify obesity, and a BMI above the 85th percentile was used to identify overweight status. This approach is appropriate for adolescents, as it accounts for the variations in BMI norms that occur with age and sex. In order not to hurt teenagers who are certainly sensitive to their physical appearance, we presented them with these tests by telling them that they were used to measure their fitness. Our goal was to use a discreet means to measure and weigh them so that they could later calculate their BMI.

### 2.4 Experimental procedure

A seven-week basketball program comprises two 50-min sessions per week, inspired by the document “Obese Student in Physical Education: An Example of Partial Aptitude”, developed by the Academic Group of Versailles in 2004. The duration of 7 weeks aligns with findings from previous studies that indicate significant behavioral and psychological changes can occur within this timeframe (28). The objective was to assess how adolescents with obesity perceive PE sessions during the basketball program, based on adapted exercises to enhance the athletic engagement and performance of students with obesity. We used exercises and the basketball methodology already described previously (22). Before the start of the experimental protocol, the participants were familiarized with the equipment. The sessions were consistently facilitated by the same instructors, ensuring consistency in timing and location.

### 2.5 Adapted intervention

In the initial sessions, the focus was on providing enjoyable activities for adolescents regardless of their fitness levels, with minimized competitiveness. As the intervention progressed, adjustments were made in 1v1 basketball drills to support students during offensive play, such as limiting the defender to one-handed play and delaying their involvement until after the attacker passed the ball. Similarly, in 3v3 drills, the game format was modified to a 3v2 setup to create more attacking opportunities. Additionally, students were permitted to rotate during offensive phases, allowing to rest more frequently and participate effectively. It's important to note that the basketball sessions were coeducational, involving both boys and girls as usual. Meanwhile, students in the control group (CG) underwent the same number of traditional basketball sessions. The intervention program followed the structure outlined in Table 2 below.

**Table 2:** Structure of the basketball intervention program for obese students.

Week	Session focus	Traditional session	Adapted session	Goal of adaptation
Week 1	Discovery	Introduction to basketball rules and basic skills	Fun activities alternating between high-intensity work and active rest, minimizing competitiveness.	Foster engagement and introduce basketball in a low-pressure environment.
Weeks 2–3	1-on-1 Drills	1-on-1 offensive and defensive play with standard rules.	Defender limited to one hand, and defender's actions delayed until attacker moves.	Reduce defensive pressure, making it easier for overweight students to participate in offensive play.
Weeks 4–5	3-on-2 Drills	3-on-3 drills with standard offensive and defensive roles	Adapted to 3 attackers vs. 2 defenders, favoring offense for overweight students.	Create more attacking opportunities for overweight students.
Weeks 6–7	5-on-5 Game	Full 5-on-5 basketball game with substitutions as desired.	Mandatory substitutions at each offensive transition to allow more frequent rest.	Reduce fatigue and provide more recovery time for overweight students.

### 2.6 Psychological measures

Before and after intervention all students completed a questionnaire to assess their perception of PE sessions The items were taken from the enjoyment/interest and perceived competence subscales of the Intrinsic Motivation Inventory, a widely used tool designed by Ryan, 1982 (43) to assess individuals' subjective experience related to a specific activity, particularly focusing on intrinsic motivation. The IMI has been validated in physical activities among adolescents (28) also with obesity (29) and in diverse cultural contexts (30). All items from these subscales were used to ensure a comprehensive assessment, in accordance with previous research validating these scales in similar settings. These were questions regarding the activity in which students with obesity had participated (e.g., “I was very interested in this activity” (interest); “I think I am quite good at this activity” (perceived competence, etc.). The students were required to respond to each statement on a 5-point Likert scale. The inventory was translated using a forward-backward translation process. Initially, a bilingual expert translated the inventory into the participants' language. Subsequently, another bilingual expert, unaware of the original wording, translated it back to ensure consistency and accuracy. After back-translation, a cultural expert made changes where necessary to improve understanding without altering the original meaning. Additionally, explanations were provided by the teaching if needed, which facilitated understanding. The aggregated subscales showed a reliable internal consistency (Cronbach's *α* = 0.84).

Prior to the first administration, a pilot test was conducted for familiarization and to identify any potential problems with the format or wording of the questionnaire. Adjustments were made based on feedback from the pilot test to improve clarity and cultural relevance.

At the end of the PE class, the questionnaires were distributed to the participants. The questionnaire was administered in a group setting to facilitate interaction and provide support among participants. Prior to administration, we provided clear instructions to ensure that all participants understood the questions. Teaching staff were available to assist with any queries during the process, further enhancing clarity and comprehension. Participants were given approximately 15–20 min to complete the questionnaire. During this time, facilitators were available to address any questions or concerns to ensure clarity.

Confidentiality of responses was strictly maintained. Only the research analyst (O.S.) had access to the individual data. Responses were anonymized before analysis, and results were aggregated to ensure participant confidentiality. The scores from the individual questionnaires were averaged for subsequent statistical analysis.

### 2.7 Basketball athletic performance evaluation

During weeks 6 and 7, which included four sessions of 5-on-5 basketball games (two sessions per week), students who were resting, recovering, or not actively participating were familiarized with the observation grid. This familiarization took place throughout the sessions, enabling the students to train in its use prior to the final peer assessment. At end of the intervention, to evaluate the individual progress of each student, a self-assessment and peer evaluation method was adopted, which aids students in honing their ability to evaluate their performance and offer constructive feedback to their peers. During an end-of-cycle test, data supporting the individual progress of each student was collected in an observation grid during a 7-minute basketball match. While some students played, effectively engaging in passing accuracy, shooting attempts, and overall gameplay skills, two student observers performed the performance evaluation. One student provided real-time comments on actions, while the other recorded pertinent information on the scorecard. The collected data was then transmitted to the respective team during strategic timeouts and at the end of the 7-min match.

### 2.8 Statistical analysis of data

Statistical analysis was conducted using the SPSS software (Statistical Package for the Social Sciences). Statistical power calculations were performed using G-power software to ensure that sample size was sufficient to detect meaningful effects and to enhance finding's reliability. Data normality was assessed using the Shapiro-Wilk test.

The Shapiro-Wilk test revealed that enjoyment/interest and perceived competence subscales of the Intrinsic Motivation Inventory (IMI) did not follow a normal distribution (*p* < 0.05).

Similarly, the athletic performance scores did not exhibit a normal distribution (*p* < 0.05), further justifying the use of non-parametric tests.

For variables that violated normality assumptions, non-parametric tests were employed. Specifically, the Kruskal-Wallis test was used to compare groups, with the effect size being calculated using *η*^2^. When significant differences were found, Dunn's *post hoc* test was applied to determine which specific groups differed. For parametric variables, Levene's test was used to confirm the equality of variances. No significant violations of homogeneity were found (*p* > 0.05), allowing us to proceed with ANOVA and paired *t*-tests where appropriate.

The analysis of ANOVA was used to evaluate pre- intervention differences between groups (EG and CG) and gender (boys and girls). Basic statistics (a paired two-sample *t*-test) were also performed to evaluate significant differences in BMI and weight between same-gender groups before and after the intervention. To assess the internal consistency of the psychological measures, Cronbach's alpha coefficient was calculated for the enjoyment/interest and perceived competence subscales of the Intrinsic Motivation Inventory (IMI).

Statistical significance was set at a probability threshold of 5% (*p* < 0.05).

## 3 Results

**Figure 1:**
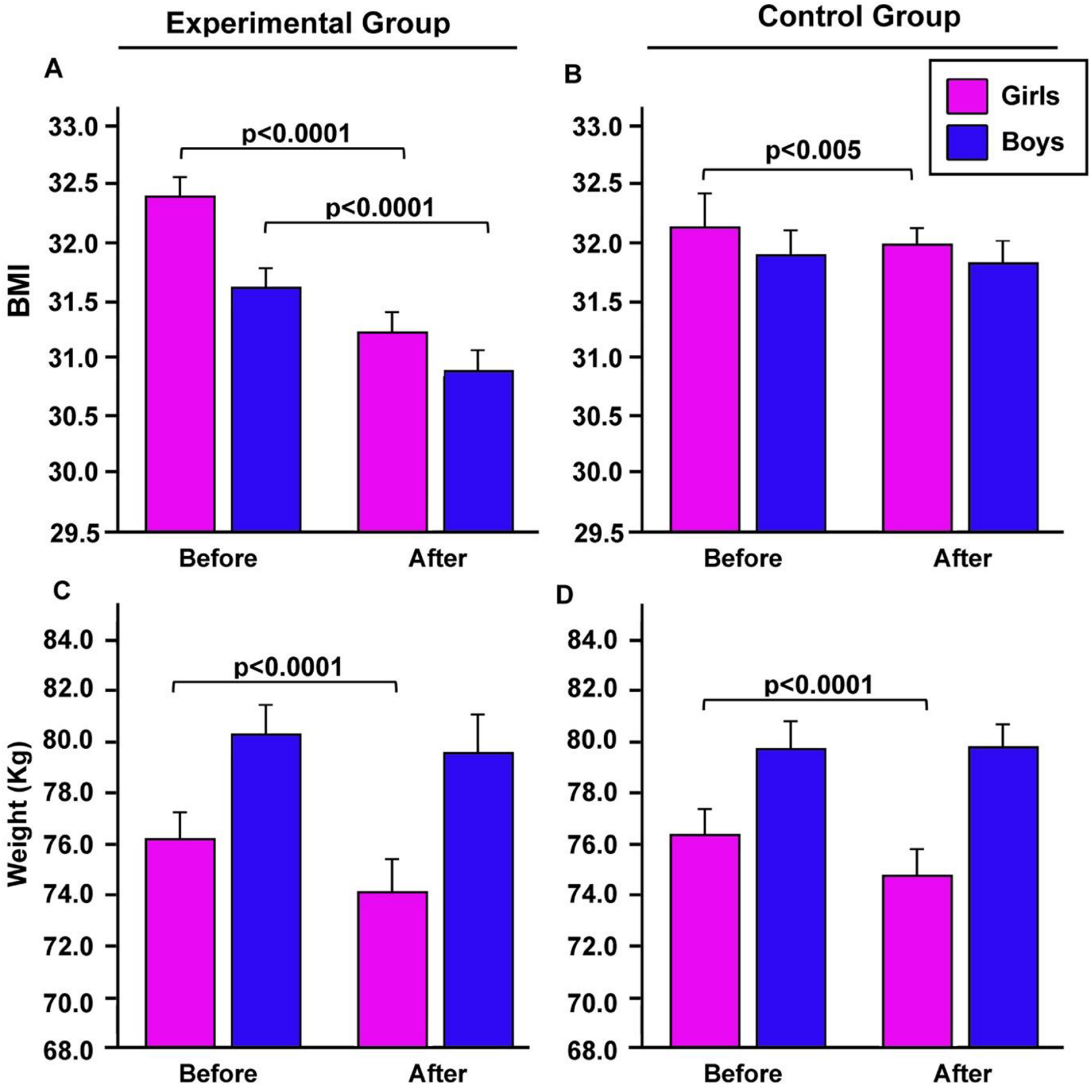
Differences in BMI and weight between the intervention **(A,C)** and control **(B,D)** groups obtained before and after intervention. *p* by paired Student's *t* test.

We firstly evaluated the effects of the adapted basketball exercises over 7 weeks period on body mass index and weight of students with obesity. After the random assignment of the students (intervention and control groups, EG and GC), no significant differences were found between the two groups, nor between girls and boys in mean values of weight, height, and BMI (Table 1, Figure 1). After the period of physical activity, there was a decrement in BMI in both boys and girls of the EG (*p* < 0.0001 by paired Student's *t*-test, *d* = 1.187, by Cohen, Figure 1A) and also in girls of the CG (*p* < 0.005, by paired Student's *t*-test, *d* = 8.087 by Cohen, Figure 1B).

Seven weeks of basketball activity also induced a decrement in body weight in the EG girls (*p* < 0.0001, by paired Student's *t*-test, Figure 1C) and in the CG girls (*p* = 0.00069, *η*^2^ = 0.4 large) compared to the initial values, i.e., before the seven weeks of basketball (Figure 1D).

### 3.1 Psychological measures

Before and after the intervention, all students' perception of physical education sessions was assessed. After the intervention, in the EG group a significant increase was obtained in interest in participation, perception of the ease of the exercise, motivation and perception of the progress achieved, compared to the values measured in the CG. In the CG, no differences emerged between before and after the intervention, for any considered parameter.

**Figure 2:**
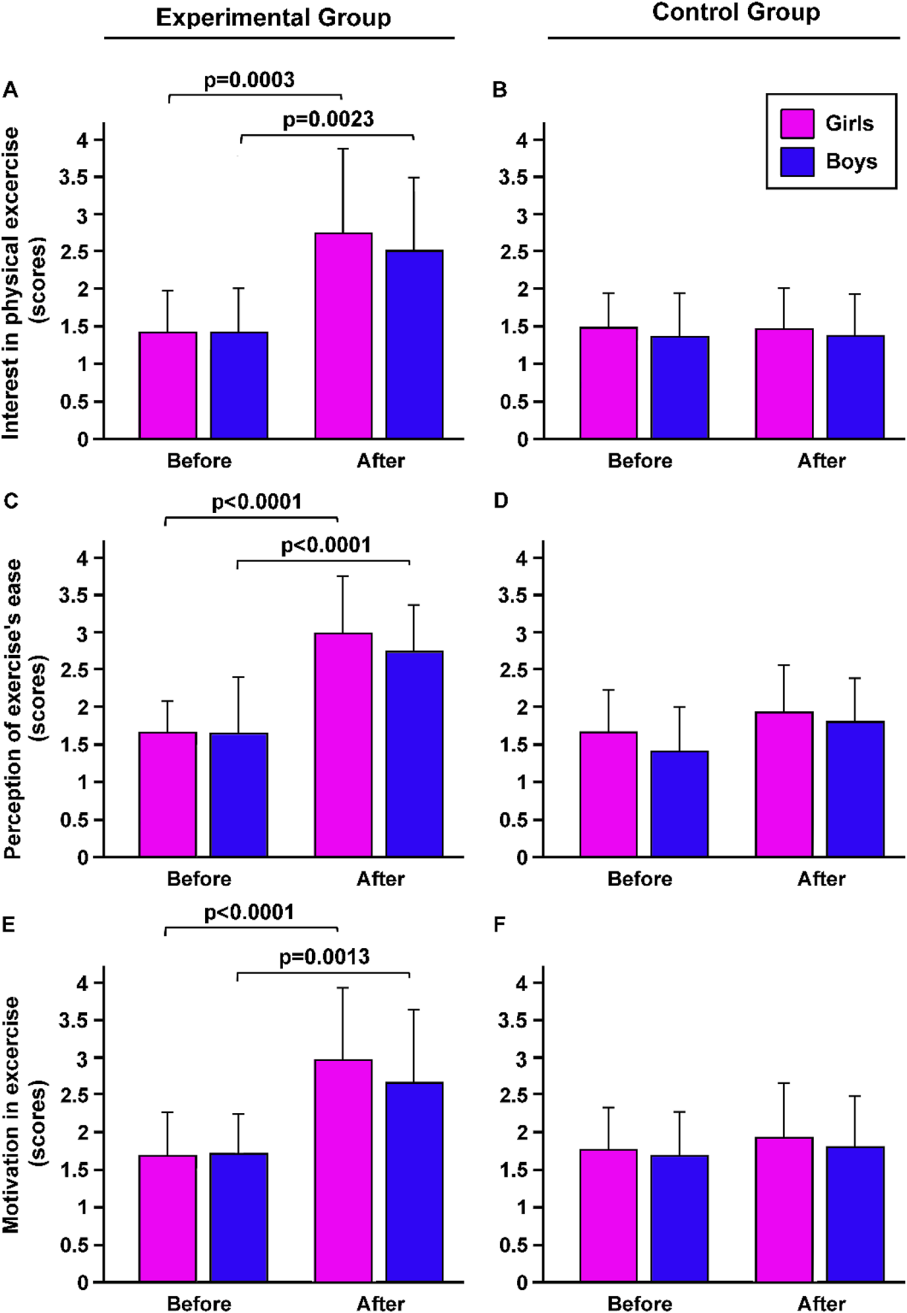
The effects of the adapted basketball program on the athletic engagement of students with obesity. Before and after the intervention, interest in participation in physical education sessions **(A,B)**, perception of exercise's ease **(C,D)**, motivation **(E,F)**, and progress achieved perception were evaluated, in both girls and boys included in EG and CG were evaluated. *p*, statistical differences assessed by by Bonferroni Dunn.

In detail, interest in physical exercise increase was observed after 7 weeks of adapted basketball cycle, in both girls (+98.8%, *p* < 0.001) and boys (+72%; *p* < 0.05) included in the EG (*p* = 0.0002889, effect size *η*^2^ = 0.19, large effect by Kruskal-Wallis's test, Figure 2A). No differences were observed between in CG (*p* = 0.9344, Figure 2B).

Statistical analysis showed that the level of perception of the ease of the exercise by the subjects in the EG was significantly higher after the adapted basketball intervention than before (*p* < 0.0001, *η*^2^ = 0.37 large; Figure 2C). In contrast, subjects in the CG showed no significant difference in perception of exercise difficulty between before and after the classic basketball cycle (*p* = 0.3138; Figure 2D).

The motivation was significantly higher after the adapted basketball cycle than before, in girls (+77%, *p* < 0.001) and boys (+56%, *p* < 0.05) included in the EG (*p* < 0.001, *η*^2^ = 0.29 large effect, Figure 2E). At the same time, no significant difference in motivation was identified in the CG before and after the classical basketball cycle (*p* = 0.8811, Figure 2F).

**Figure 3:**
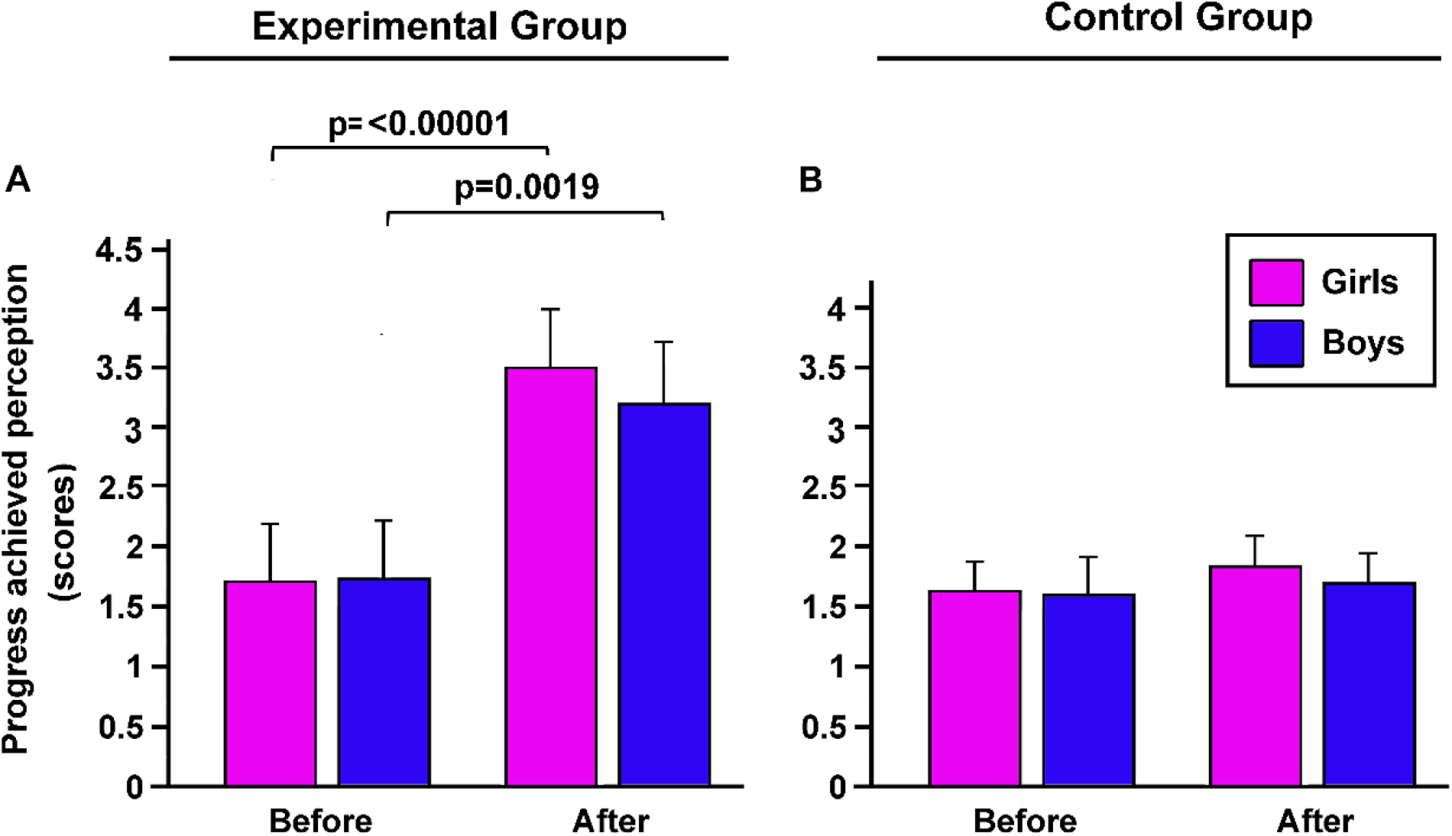
The effects of the adapted basketball program on perceived competence of students with obesity. Before and after the intervention, perception of improvement in competence were evaluated, in both girls and boys included in experimental **(A)** and control group **(B)** were evaluated. *p*, statistical differences assessed by Bonferroni Dunn.

Our results indicate that gender does not influence the perceived competence of the students who, after the adapted basketball cycle, all perceived themselves as having higher competences than before the training program (*p* = 0.0001, *η*^2^ = 0.48 large, Figure 3A).

Instead, no significant difference was obtained in the CG regarding the perceived competence before and after the classical basketball cycle (*p* = 0.8742, Figure 3B).

**Figure 4:**
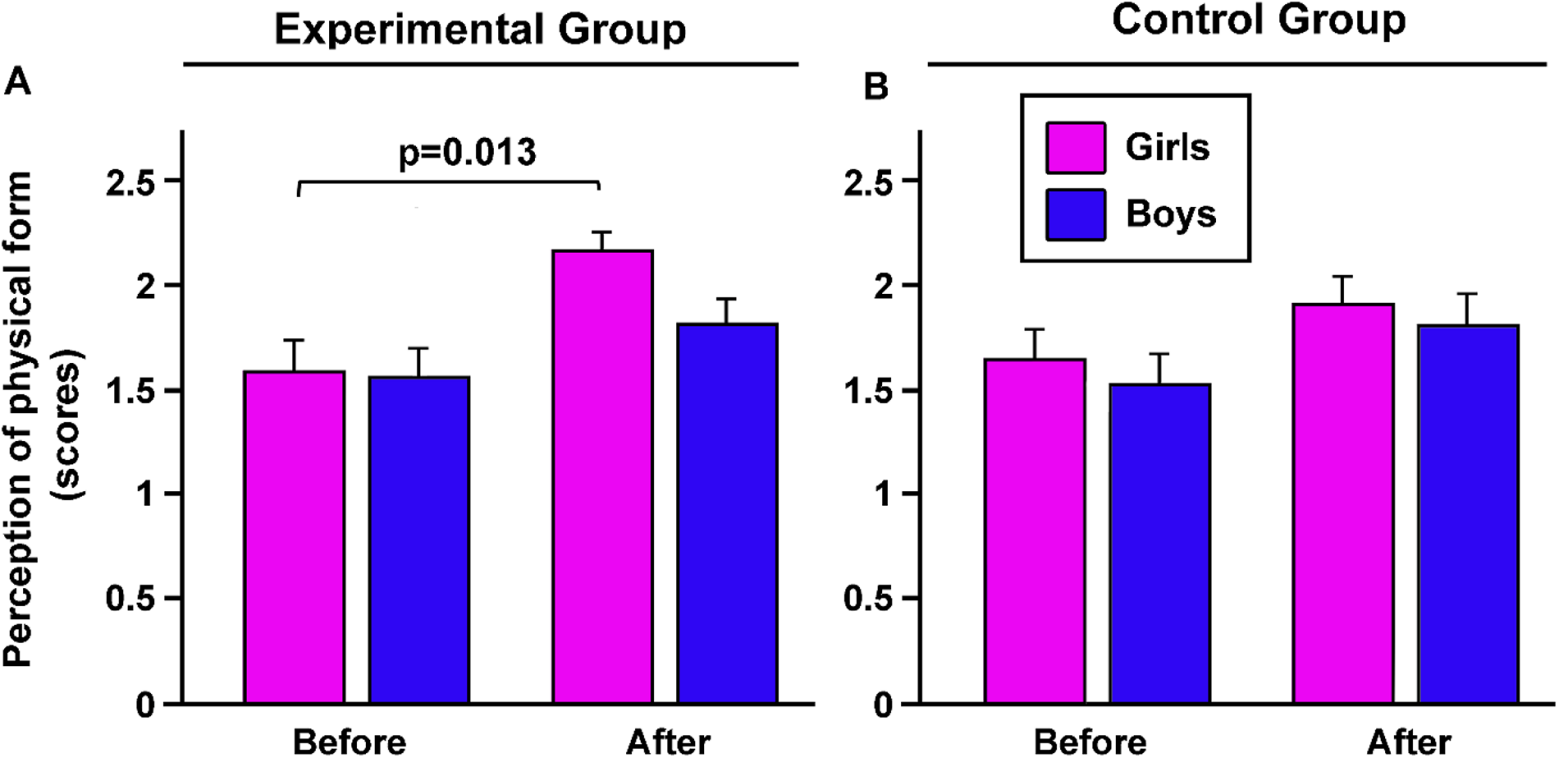
The effects of the adapted basketball program on the perceived physical form of students with obesity. Before and after the intervention, perceived physical form was evaluated, in both girls and boys included in experimental **(A)** and control group **(B)** were evaluated. *p*, statistical differences assessed by Bonferroni Dunn.

The perceived level of physical form significantly increased in girls of the EG after the adapted basketball cycle (*p* = 0.013; Figure 3A). Conversely, no significant difference was found in the perception of physical form among boys of the EG and both genders from the CG (*p* = 0.784, Figure 4).

### 3.2 Athletic performance in basketball

We evaluated the number of passes made during a 7-minute basketball match in players of both EG and GC. Although, the results were not statistically significant, we observed that 53% of the passes were failed, while 47% of the passes were successful in both groups.

**Figure 5:**
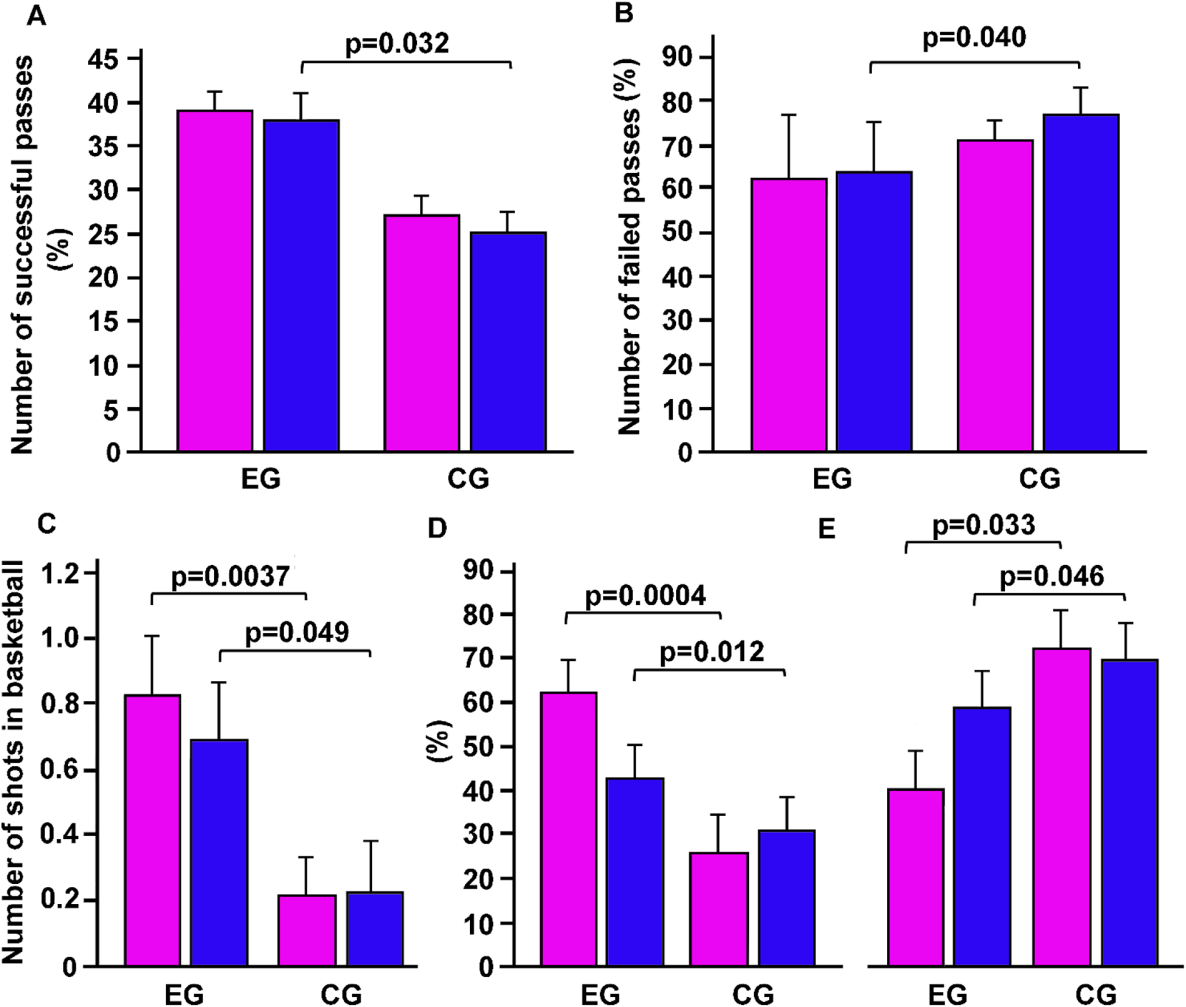
The effects of the adapted basketball program on athletic performance in basketball of students with obesity. After the intervention, both girls and boys included in the experimental (EG) and control group (CG) were evaluated for the number of successful passes **(A)**, failed passes **(B)**, shots made **(C)** successful shots **(D)** and failed shots **(E)** p, statistical differences assessed by Bonferroni Dunn.

However, more successful passes (*p* = 0.032, *η*^2^ = 0.042, small effect, by Kruskal-Wallis's) and fewer failed passes (*p* = 0.040, *η*^2^ = 0.023, small effect,) were observed in boys from the EG compared to those from the CG (Figures 5A,B). Regarding shots, we observed a higher average number of basketball shots per student in the EG group compared to the CG group (*p* = 0.0051; *η*^2^ = 0.32, indicating a large effect, Figure 5C). This difference was particularly pronounced among girls, with a *p*-value of 0.0037 and an *η*^2^ of 0.43, also indicating a large effect (Figure 5C). These findings suggest that the adapted basketball exercises were particularly beneficial for girls. This highlights the importance of personalized programs that can more effectively meet gender needs or motivational factors. In addition, girls from the EG demonstrated a greater number of successful shots and fewer failed shots compared to the CG (*p* = 0.0004 and *p* = 0.033, respectively). Similarly, boys from the EG also showed more successful shots and fewer failed shots compared to the CG (*p* = 0.012 and *p* = 0.046, respectively) (Figures 5D,E).

## 4 Discussion

This study aimed to address the following question: *Does an adapted basketball-focused approach improve the performance and athletic engagement of students with obesity*?

According to the initial hypothesis, students with obesity, included in the EG, perceived adapted physical activity as easier and more motivating, compared to those in the CG and undergoing a classical basketball cycle.

Although, managing obesity requires a multi-faceted approach, it is widely recognized that physical activity is a principal intervention in addressing the increasing prevalence of obesity in the pediatric population. Alongside physical activity, critical interventions such as dietary changes and behavioral modifications are essential for effective obesity prevention and treatment. These strategies can help mitigate associated health problems, including metabolic syndrome, poor physical health, mental disorders, respiratory issues, and glucose intolerance, which may persist into adulthood (6, 46, 47). Positive effects of high-intensity interval training on body composition, cardiovascular parameters, and cardiorespiratory fitness among adolescents have been extensively described (48–50). Unfortunately, the decrease in participation in physical activity during adolescence suggests that there are deficiencies in current physical education programs; furthermore, youth with obesity often have poorer motor skills and physical condition than students of normal weight (27, 28), thus encountering many difficulties during PE sessions.

This leads them to perceive a lack of interest and motivation, low self-esteem, incompetence, poor body image, and avoidance of physical exercise (29, 30), Therefore, students with obesity need a supportive environment around them, especially among their friends and teachers, free from stigma or negative attitudes (51). Previous studies indicating that adapted PE classes could be an effective strategy to improve the inclusion of young with obesity or overweight and a means to prevent obesity (20, 22, 37, 47). In this context, and in the belief that physical activity interventions are useful for preventing or treating obesity, we here suggested a basketball training activity, since basketball practice improve the body composition of adolescents with obesity (8, 52, 53), players' physical performance and motor skills (8), and it can reduce the negative emotional state, thus effectively improving the mood of adolescents with obesity (54). With the aim of facilitating overcoming the obstacles encountered by young people with obesity when undertaking physical activity (55), we have adapted basketball exercises to promote socialization and fun, and therefore favorably influencing the regulation of emotions and empathy (21, 22, 56).

Here we demonstrate that students appreciated this adapted basketball program which indeed improved motivation, interest, and positive perception of the sessions, providing them with the opportunity to learn like their peers, even in the presence of reduced initial physical abilities. Indeed, active participation in learning situations adapted to students' characteristics allows them to grasp the knowledge and skills necessary for simultaneous progress in motor and methodological effectiveness (57).

Since perceived competence provides the means to modify one's behaviors (58), it is worth highlighting the importance that, after the adapted basketball cycle, students with obesity perceived greater competence regarding the exercises. It is known that individuals with higher perceived competence in PE are more likely to participate in PA at school (59) and outside (60).

As is well known, competence is one of many determinants of enjoyment of and participation in physical activity (44). Thus, improving students' views of their physical abilities is an important goal, particularly among adolescents with obesity who show low perceived competence. Furthermore, the interaction between gender, competence, and enjoyment should be a key consideration in planning the content and delivery of PE (45).

While our intervention did not result in significant gender differences in perceived competence, it is noteworthy that girls exhibited greater improvements in perceived physical form. This observation may be partially explained by research suggesting that adolescent girls tend to be more self-conscious about their body image and physical appearance compared to boys (61). Studies reveal that girls often experience greater societal pressure regarding their appearance, which may heighten their sensitivity to changes in physical form during interventions aimed at physical health improvement (62, 63). Furthermore, it has been suggested that interventions promoting physical activity can help reduce body dissatisfaction in girls, potentially leading to the observed increase in their perceived physical form (64, 65).

On the contrary, if students perceive their physical abilities as not amenable to improvement through practice, it is unlikely that interest and motivation will be increased in PE settings (66). The adapted basketball cycle had a large and substantial impact on students' perceived competence. Essentially, this indicates that the intervention was very effective in improving how students with obesity perceive their physical abilities; this is important because perceived competence is related to continued participation in physical activity. By feeling more competent, students feel more competent and are therefore more likely to engage in future physical activity both inside and outside of school.

Although gender differences in perceived competence in physical education have been consistently reported (45) here we did not find a significant gender difference. Our intervention design was likely inclusive and engaging for both genders equally in terms of competence-related activities. On the other hand, gender stereotypes and social expectations regarding competence may play a role in limiting perceived differences in this specific measure.

Overweight and obesity status impact children and adolescents' physical self-perception (67), especially when considering girls (68), who tend to have greater dissatisfaction with their body image (69).

The very low level of physical self-perception measured in both girls and boys before the intervention is concerning since physical self-perception is related to physical activity, motor competence, and performance during adolescence (70).

Furthermore, with lower perceptions of their physical ability, overweight children and adolescents had significantly lower physical competencies than their normal-weight peers (71), which could lead to disengagement in physical activity and lower physical activity levels (72). The less an individual engages in physical activity, the more his physical competence decreases causing the reduction of his self-perceptions (70).

Our adapted PE program could, especially in girls, allow us to decrease this negative spiral of disengagement in physical activity, as a result of the improvement in perceived physical competence after the intervention. Girls may then benefit psychologically, perhaps related to improvements in body image; this favors more continued participation. It was demonstrated that obesity significantly hinders some motor activities, such as jumping, climbing, or squatting. In addition, in adolescents, there appears to be an inverse relationship between physical fitness and BMI level, with cardiorespiratory endurance (73). In some types of aerobic activities, such as running, overweight adolescents are unable to cover the same distance as their peers with a normal weight, as they become tired more quickly (36). Also, for this reason, the adaptation of exercises seems to be the desired solution since the student with obesity having sometimes reduced motor skills, is unable to take part in some sporting activities (74). Active participation in learning situations adapted to students' characteristics allows them to grasp the knowledge and skills necessary for simultaneous progress in motor and methodological effectiveness (57).

Here, after 7 weeks of adapted basketball exercises, EG students with obesity showed few missed shots and a large number of successful shots at the basket. Therefore, although a significant difference was observed in the number of completed and missed passes during a five-on-five match (which served as an end-of-cycle test) between the boys of both groups, the number of failed passes remained high. We note that negative reinforcement of the attempted pass often results in a reluctance to take the next pass, and the long-term effect may be that the player does not enjoy the PE. Thus, following a standard basketball cycle, students did not improve their athletic performance. Various factors contribute to insufficient academic performance including students' difficulties in adapting to school life and tasks imposed by the educational system (related to the learning process, school rules, or interaction with peers and teachers) (75). The results of this study also demonstrate that some different levels of academic maladaptation can be effectively addressed and overcome through the adaptation of educational contents. Our type of adaptation, according to the “EPS adapted and EPS & disability” commission of the Academy of Versailles, enables students with obesity to develop a positive relationship with their bodies, regardless of physical limitations and it also contributes to enhancing sports performance in basketball.

Several confounding variables could influence the results of this study. First, the sample size, particularly when split by gender, may limit the generalizability of the results. A larger sample could enhance the statistical power and provide more robust conclusions; further studies with larger populations could definitively confirm these results. Furthermore, pre-existing differences in baseline physical activity levels could have influenced the results, as students who were more active before the intervention may have improved more. This factor was not controlled for, but we relied on self-reported activity levels. Second, different psychological states, such as pre-existing levels of motivation, concerns about body image, or self-esteem, could also have influenced the extent of improvement, particularly in perceived fitness among girls. These psychological factors were also not fully accounted for and could have influenced the results. In addition, we can assume that social support from peers, teachers, or family members may contribute to differences in engagement and performance, as greater support may lead to greater participation. Finally, the seven weeks of intervention may not have been sufficient to observe more significant long-term changes, this may actually limit the generalizability of the results. It is therefore desirable to carry out future studies that should consider longer intervention periods and, also, include larger and more diverse samples with the aim of mitigating these limitations and exploring the sustainability of these positive effects.

## 5 Conclusion

Our study demonstrates the effectiveness of an adapted basketball program in improving athletic engagement and performance among adolescents with obesity. The tailored exercises increased motivation, perceived competence, and physical fitness, particularly in girls. This highlights the importance of personalized physical education (PE) interventions in promoting positive experiences and skill development for students with obesity. By adapting PE courses, students with obesity acquire essential skills while learning alongside their peers, which fosters a more inclusive learning environment. The findings also address a broader challenge experienced by educators worldwide: declining physical health and insufficient participation in physical activity among adolescents. While factors such as individual interest, motivation, and physical and psychological well-being have rarely been studied in PE settings, our research offers a strategy that aims to improve these outcomes among students with obesity. The adapted basketball program effectively improves students' perception of PE sessions, and increases their interest, motivation, and sense of progress, regardless of gender or body type. From a practical perspective, these findings may be relevant to educational settings, in that teachers can implement similar adaptive programs to integrate students with obesity into physical activities in a safe and supportive manner. This not only helps prevent obesity, but encourages sustained physical activity, reducing stigma and promoting inclusion. Finally, future research should investigate the long-term effects of these interventions to assess whether the observed benefits are sustainable. Additionally, the applicability of adapted exercises in other sports could be explored to provide further insights into optimizing physical education programs for students with different physical and psychological needs.

## Data Availability

The original contributions presented in the study are included in the article/Supplementary Material, further inquiries can be directed to the corresponding authors.
